# Persistence, impacts and environmental drivers of covert infections in invertebrate hosts

**DOI:** 10.1186/s13071-017-2495-8

**Published:** 2017-11-02

**Authors:** Inês Fontes, Hanna Hartikainen, Chris Williams, Beth Okamura

**Affiliations:** 1 Department of Life Sciences, Natural History Museum, Cromwell Road, London, SW7 5BD UK; 2 Scottish Fish Immunology Research Centre, Aberdeen University, Aberdeen, AB24 2TZ UK; 3 EAWAG, Department of Aquatic Ecology, Überlandstrasse 133, CH-8600 Dübendorf, Switzerland; 4 ETH Zürich, Institute of Integrative Biology (IBZ), Zürich, Switzerland; 5 Environment Agency, National Fisheries Laboratory, Brampton, Cambridgeshire PE28 4NE UK

**Keywords:** *Tetracapsuloides bryosalmonae*, *Fredericella sultana*, Proliferative kidney disease, Myxozoans, Bryozoans, Productivity, Temperature, Host condition-dependent effects, Covert infection strategies, Fish disease reservoirs

## Abstract

**Background:**

Persistent covert infections of the myxozoan, *Tetracapsuloides bryosalmonae*, in primary invertebrate hosts (the freshwater bryozoan, *Fredericella sultana*) have been proposed to represent a reservoir for proliferative kidney disease in secondary fish hosts. However, we have limited understanding of how covert infections persist and vary in bryozoan populations over time and space and how they may impact these populations. In addition, previous studies have likely underestimated covert infection prevalence. To improve our understanding of the dynamics, impacts and implications of covert infections we employed a highly sensitive polymerase chain reaction (PCR) assay and undertook the first investigation of covert infections in the field over an annual period by sampling bryozoans every 45 days from three populations within each of three rivers.

**Results:**

Covert infections persisted throughout the year and prevalence varied within and between rivers, but were often > 50%. Variation in temperature and water chemistry were linked with changes in prevalence in a manner consistent with the maintenance of covert infections during periods of low productivity and thus poor growth conditions for both bryozoans and *T. bryosalmonae*. The presence and increased severity of covert infections reduced host growth but only when bryozoans were also investing in the production of overwintering propagules (statoblasts). However, because statoblast production is transitory, this effect is unlikely to greatly impact the capacity of bryozoan populations to act as persistent sources of infections and hence potential disease outbreaks in farmed and wild fish populations.

**Conclusions:**

We demonstrate that covert infections are widespread and persist over space and time in bryozoan populations. To our knowledge, this is the first long-term study of covert infections in a field setting. Review of the results of this and previous studies enables us to identify key questions related to the ecology and evolution of covert infection strategies and associated host-parasite interactions.

**Electronic supplementary material:**

The online version of this article (10.1186/s13071-017-2495-8) contains supplementary material, which is available to authorized users.

## Background

Covert or latent infections are non-infective, persistent forms of a parasitic infection that are asymptomatic in host populations [1]. Stages causing covert infections may be latent or may undergo low levels of replication [2]. When activated, covert infections become overt and result in detectable diseases [3] that may be lethal [4]. Covert infections are typically caused by agents whose small sizes enable persistence as cryptic stages within much larger hosts without invoking obvious harm. Covert viral infections have received most attention, for example those causing human immunodeficiency virus (HIV) [5] and hepatitis C [6] in humans, and those associated with insect diseases, such as granuloviruses (GV) [7], baculoviruses [8] and nucleopolyhedroviruses [9]. Bacteria can also cause covert infections in many organisms, including humans (e.g. tuberculosis), fish [10] and plants [11]. Eukaryotic organisms causing covert infection include the microsporidian, *Encephalitozoon cuniculi* [12], and protozoans, such as *Plasmodium falciparum* [13]. At present, however, we are aware of only a single group of metazoans that demonstrate covert infection dynamics - myxozoans that are associated with freshwater bryozoan [14] and annelid [15] hosts. Myxozoans are a clade of endoparasitic cnidarians with complex life-cycles, exploiting invertebrates and vertebrates as primary and secondary hosts, respectively. Morphological simplification and miniaturisation, along with a capacity for multiplication within hosts, have enabled myxozoans uniquely to converge on infection strategies associated with microparasites [16]. Covert infections of malacosporean myxozoans in freshwater bryozoans (benthic, colonial invertebrates) have been examined in two systems: *Tetracapsuloides bryosalmonae* in the bryozoan *Fredericella sultana* [17–19] and *Buddenbrockia allmani* in the bryozoan *Lophopus crystallinus* [20]. The former system has been particularly investigated as *T. bryosalmonae* is the causative agent of proliferative kidney disease (PKD) of salmonid fish. It has been argued that covert infections that cause little to no adverse effects enable long term persistence of *T. bryosalmonae* in bryozoan populations and hence a disease reservoir for fish [14, 21]. Confirmation that infected overwintering stages of bryozoans (statoblasts) are viable [22, 23] reveals one mechanism for such infection persistence in bryozoan populations. In addition, transmission of *T. bryosalmonae* to fish upon exposure to colonies deriving from infected statoblasts [22] indicates that infection of statoblasts creates an effective disease reservoir. However, observations and collections of live colonies of *F. sultana* during winter periods [18, 24] suggest that covert infections might persist in bryozoan colonies throughout the year. If so, overwintering colonies may offer an additional and direct means of infection persistence within populations and this could also contribute to the disease reservoir for fish.

Molecular diagnostics have shown that *T. bryosalmonae* infections persist over weeks to months at varying prevalences in bryozoan populations. One study found prevalences to range from 0 to 53% during early April to mid-September in three bryozoan populations situated along a short (~15 m) stretch of the River Cerne (Dorset, UK) [18]. A second study revealed infection prevalences of 0–17% in bryozoans transplanted into a single locality on the River Itchen (Hampshire, UK) during late June to early August [25]. Neither study distinguished covert from overt infections. How covert infection prevalences vary over a full annual cycle in bryozoan populations remains unknown, and variation in prevalences in bryozoan populations within and between rivers is poorly understood. Furthermore, previous studies used polymerase chain reaction (PCR) primers that are likely to have underestimated infection prevalences due to lower sensitivity than that of more recently designed primers.

To improve our understanding of the dynamics, impacts and implications of covert infections in bryozoan populations we utilised a recently developed and highly sensitive PCR assay to address the following hypotheses: (i) Bryozoan hosts harbour covert infections of *T. bryosalmonae* throughout the year, contributing to the disease reservoir for fish; (ii) Covert infection prevalences and severities vary within and between rivers; (iii) Covert infection prevalences and severities are influenced by environmental conditions; and (iv) Covert infections have no impact on bryozoans in the field, a prediction in keeping with limited evidence of little to no effect on host growth and statoblast production in laboratory studies (see below).

Freshwater bryozoans are colonial, suspension feeding invertebrates that are ubiquitous but overlooked residents of freshwater environments. In temperate regions, colonies grow during warmer months of the year by budding new zooids, each of which has a tentacular crown (the lophophore) used for suspension feeding [26]. The most common bryozoan host of *T. bryosalmonae* is *F. sultana,* which can form dense stands of colonies in the interstices of submerged roots of riparian trees [18]. *Fredericella sultana* grows as branching, tubular colonies and reproduces mainly asexually by colony fragmentation and the production of over-wintering, dormant seed-like stages called statoblasts [27]. Statoblasts enclose germinal tissues and hatch to give rise to small colonies in spring as temperatures increase. *F. sultana* can also persist during winter as live colonies [24].

*Tetracapsuloides bryosalmonae* cycles between two developmental stages within bryozoans, resulting in covert and overt infections. The former are characterised by cryptic stages consisting of single cells associated with the body wall [17] and are detectable by PCR. Controlled laboratory studies provide evidence that covert infections are largely avirulent. Thus, for bryozoans collected from one river system (the River Cerne [28]) there were no detectable effects of covert infection on host growth, except when hosts were also investing in statoblast production, nor in the propensity to produce statoblasts. In addition, the number of statoblasts produced was not impacted by whether colonies were covertly infected in bryozoans originating from two other river systems (the Rivers Avon and Dun [29]). Furthermore, infected statoblasts from three river systems (the Rivers Itchen, Dun and Lyssbach) have been shown to be viable [22, 23] and to exhibit greater hatching success than uninfected statoblasts [29]. Covert infections are also present in colony fragments that detach from parental colonies in the field [29]. Both statoblast production and colony fragmentation are modes of host propagation that *T. bryosalmonae* can exploit to effectively achieve vertical transmission to new bryozoan colonies. Overt infections involve the development of multicellular sacs that are readily observed with a stereomicroscope circulating freely within the body cavity (e.g. [19, 28, 30]). Spores mature within sacs and are released into the water to infect fish. Overt infections are virulent, impacting bryozoan growth [28] and causing temporary castration by inhibiting statoblast production [31]. Overt infection prevalences are high in late spring and autumn [18, 19] when the hosts are in good condition [19, 28] and able to support the development of these relatively large, rapidly growing and energetically costly stages [28, 31]. Sac production in highly stressed, food-deprived bryozoans [29] demonstrates that overt infections can also develop in response to potential host death, a form of terminal investment. This response may explain infection of fish in winter [32] when conditions for bryozoans are poor.

## Methods

### Study sites and sample collection

*Fredericella sultana* colonies were collected every 45 days over 1 year from three rivers in southern England: the River Avon (near Bickton, Hampshire), the River Dun (Hungerford, Berkshire) and the River Itchen (Winchester, Hampshire). The Rivers Avon and Dun were sampled for 12 months beginning in October 2012, staggering the dates between sites to accommodate processing. The River Itchen was sampled for 12 months beginning in October 2013. The sites were chosen based on the presence of PKD on fish farms associated with the rivers (O. Robinson, pers. comm.) and the presence of *F. sultana* populations on submerged tree root systems (B. Okamura, pers. obs.). Up to 100 branches of bryozoan colonies (depending on availability) were collected haphazardly using forceps from each of three tree root systems in each river. Additional file 1: Table S1 provides locations of the tree root systems sampled in each river. Branches detached from colonies were placed into individual 15 ml plastic tubes filled with river water and were kept at 4 °C for 24 h until dissection (see below).

### Environmental variables

Temperature, flow speed and dissolved oxygen (DO) were recorded on almost every sampling trip. Temperature and DO were measured in each river at the root located furthest downstream using an oximeter (WTW Oxi330). To characterise incoming flow experienced by the root system, flow speed was measured in a position directly next to the initial development of the root system and at one third of the river depth from the surface using an open channel electromagnetic flow meter (Valeport 8008/801) and positioning the flat flow sensor in an upstream direction to estimate mean speed of flow (over 60 s). Flow was recorded at the same position and equivalent depths during each sampling trip. During the last four sampling trips (i.e. from April onwards) samples were taken for water chemistry, productivity, turbidity and conductivity analyses (Additional file 2: Table S2) next to the root located furthest downstream in each river (except in the River Itchen where samples were taken from root 2) according to standard procedures. The water samples were stored at 4 °C and analysed within 24 h by the Environment Agency’s National Laboratory Service (NLS), Starcross, UK.

### Colony attributes and infection state

Detached branches (henceforth referred to as colonies) were observed using a stereomicroscope to determine their status, i.e. whether they were alive or dead (no living zooids) and in both cases, whether they contained statoblasts. The sizes of a subset of live colonies (numbers counted according to time permitting and ranging from 41 to 87 colonies per root system) were determined by counting the total number of live zooids (lophophores and digestive tracts present). All colonies were then dissected (using dissection tools sterilised with ethanol) to determine whether mature statoblasts and overt infections (sacs) were present. Statoblasts were considered to be mature if they had assumed the typical brown colouration due to tanning of chitin. The sizes of dead colonies that contained mature statoblasts were recorded as zero. Following dissection, colonies were preserved in 100% ethanol and stored at -20 °C (with the exception of dead colonies). Data on colony size (excluding dead colonies) and statoblast production were used to analyse the impacts of covert infection on bryozoans in each river system.

Colonies that were not overtly-infected were screened using PCR to characterise proportions of uninfected and covertly-infected colonies. The number of colonies screened by PCR was determined by availability or, when sufficiently numerous, by haphazardly choosing subsets of colonies from each root (up to 55 colonies per root). DNA was then extracted from each bryozoan colony (i.e. the tissue present in detached branches) using a modified hexadecyltrimethylammonium bromide (CTAB) protocol (see Additional file 3). Covert infections were detected using a PCR assay with cycling conditions as in Hartikainen et al. [23] based on primers diagnostic for *T. bryosalmonae* 18S rDNA [514F_new (5′-ATT CAG GTC CAT TCG TGA GTA ACA AGC-3′) and 776R (5′-CTG CCC TTA ATT GGG TGT ATC AGC-3′)] to produce an amplicon of 244 bp. This set of primers appears to be highly sensitive to *T. bryosalmonae*, indicating much greater infection prevalences in bryozoan populations than those revealed by different primers used in previous studies [23]. The PCR products (3 μl) were run on a 1.5% agarose electrophoretic gel. Infection intensity was characterised by the PCR product’s molecular weight (ng/μl) estimated by the gel band intensity of two individual replicate PCRs [29]. This is a semi-quantitative method of estimating colony infection intensities (precluding accurate estimates of very strong/weak signals [29]). In view of this potential constraint we amplified and gel-analysed all samples in duplicate and prepared, ran, photographed and analysed gels using identical conditions [29]. Infection severity was then calculated by dividing infection intensity by colony size.

One sample that was positive for *T. bryosalmonae* for each root in each river was selected randomly (total of 9 samples) and verified by direct sequencing using an ABI PRISM® 3700xl DNA analyser (Applied Biosystems, Foster City, USA) and BigDye v1.1 chemistry. Bryozoans were retained for 24 h prior to dissection which allowed voiding faeces (and hence ingested spores) and promoted breakdown of any *T. bryosalmonae* spores retained on surfaces or in the water (spores degrade between 12 and 24 h, as shown in [33]). Dishes were rinsed with warm water and dissection equipment with ethanol between colonies. We also employed negative controls in DNA extractions and in PCR runs.

### Statistical analysis

All statistical analyses were conducted using R version 2.15.1 [34]. Principal components analysis (PCA) was performed on the environmental variables, circumventing the problem of multi-collinearity, to create uncorrelated axes and to investigate variation associated with rivers by cluster analysis. PCA was run on centred and scaled variables (i.e. mean = 0 and standard deviation = 1) and calculated from the correlation matrix using singular value decomposition (SVD) with the *stats* package version 3.1.1 [34]. PC biplots and ellipses with normal probability contours set to 68% were created using the *ggbiplot* package version 0.55 [35]. A Scree plot was used to choose the number of principal components (PCs) or axes to consider as meaningful representations of the data and to include as fixed explanatory variables in subsequent Generalised Linear Mixed Models (GLMMs).

Differences in live colony size were compared across sampling trips using a Generalised Linear Model (GLM) with a Quasi-Poisson error distribution. The proportion of colonies producing statoblasts (both live and dead) was compared amongst rivers using a GLM with a binomial error distribution.

Relationships between infection state and explanatory variables, including environmental variables and colony attributes, were assessed with GLMMs following the methods described in Zuur et al. [36]. Infection measures of colonies used as response variables included covert infection status (present or absent) and, conditional on covert infection being present, the infection severity. We used the package *lme4* version 1.1-7 [37] assuming a binomial error distribution to analyse infection status, and the package *nlme* version 3.1-117 [38] to analyse infection severity. Variability in the response variable amongst rivers, roots and sampling trips was investigated graphically. Random effects models were then built to determine the optimal random structure using restricted maximum likelihood estimation (REML) and maximum likelihood estimation (ML) for infection severity and status, respectively. Univariable analyses were used to explore the influence of each fixed explanatory variable (Table 1). Only those variables with *P*-values < 0.25 were included in a maximal model using ML estimation following a visual inspection of the data. Variables and interactions were then removed from the maximal model in a stepwise fashion by establishing whether their removal caused a significant change in the model’s Akaike Information Criterion (AIC) value.

**Table 1:** The suite of fixed explanatory variables relating to environmental measurements and bryozoan colony characters that were incorporated in mixed model statistical analyses

Category	Explanatory variable
Water chemistry	Dissolved oxygen as O_2_ (mg/l)
	Chemical Oxygen Demand (COD) (mg/l)
	pH
	Alkalinity to pH 4.5 as CaCO_3_ (mg/l)
	Hardness, total as CaCO_3_ (mg/l)
	Ammonia un-ionised as N (mg/l)
	Ammonia nitrogen as N (mg/l)
	Nitrate as N (mg/l)
	Nitrite as N (mg/l)
	Nitrogen, total oxidised as N (mg/l)
	Orthophosphate, reactive as P (mg/l)
	Magnesium (mg/l)
	Calcium (mg/l)
	Chloride (mg/l)
	Cadmium (mg/l)
Productivity	Bacterial Oxygen Demand (BOD) (mg/l)
	Chlorophyll a, acetone extract (μg/l)
	Total confirmed coliforms, membrane filtration (CFU/0.1 l)
	Total presumptive coliforms, membrane filtration (CFU/0.1 l)
Other environmental factors	Water temperature (°C)
	Water flow (m/s)
	Turbidity (FTU)
	Conductivity at 25 °C (μS/cm)
Colony characters	Zooid number (colony size)
	Statoblast presence

In view of our broad range of results the associated statistical tests and their inferences are incorporated collectively in table format to enable ready comparison and summary.

## Results

### Environmental variables

Temperatures in the three rivers generally declined from October to December and remained low until early spring. Temperatures in the River Dun did not vary as much as in the other two rivers (Additional file 4: Figure S1). The results for all environmental variables (except temperature) and for flow speed are provided in Additional file 2: Table S2 and Additional file 5: Table S3, respectively. Data for 21 environmental variables, including temperature and flow speed, measured for the last four sampling trips to the Rivers Avon and Dun were suitable for PCA (the large amount of missing data for the River Itchen precluded its use). Two PCs explained 81% of the variance in the data - PC1 which represents the two rivers explained some 56% of the variance and PC2 which represents the four sampling trips explained some 25% of the variance (see Additional file 6: Figure S2 and Additional file 7: Table S4)*.* PC1 had strong positive loadings for Bacterial Oxygen Demand (BOD), nitrogen associated variables, chloride, orthophosphate, magnesium, coliforms (presumptive and confirmed) and temperature, and strong negative loadings for DO and alkalinity. PC2 had strong positive loadings for chlorophyll-a and turbidity, and strong negative loadings for temperature, alkalinity, orthophosphate, conductivity, hardness and calcium.

Ellipses and colour coded data demonstrate that rivers and sampling trips are not overlapping in the PC space (Additional file 6: Figure S2). These results show that rivers differ in water chemistry and that this varies less between sampling trips 6–8 on the River Avon.

### Bryozoan hosts over time and space

**Fig. 1:**
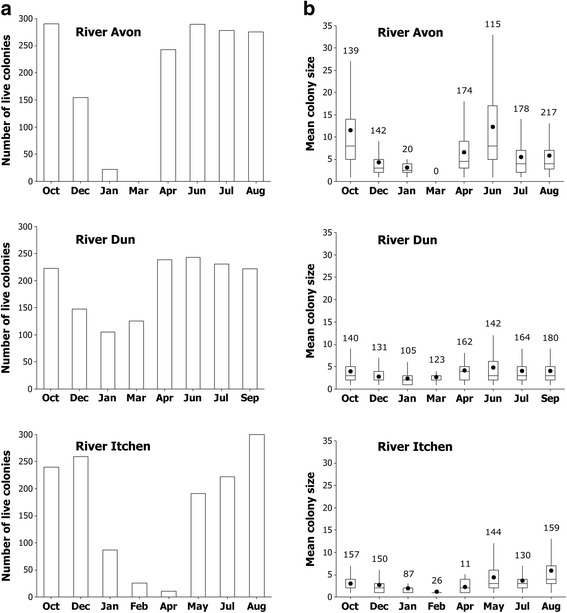
Variation in numbers of live colonies and colony size over time. **a** The total number of live colonies collected every 45 days over 12 months from the Rivers Avon, Dun and Itchen (pooling over the number of colonies sampled from the three tree root systems in each river). **b** Boxplot showing mean (black circle) colony size (total number of zooids per live colony) for a subset of colonies sampled in each river over time. Numbers are *n*-values (pooling colonies from the three tree root systems in each river and excluding dead colonies with statoblasts)

Up to 300 *F. sultana* colonies were collected during each of eight sampling trips from the Rivers Avon (total number of colonies 1563), Dun (total number of colonies 1562) and Itchen (total number of colonies 1337) (Table 2, Fig. 1a). The majority of the colonies collected on each sampling date were alive but the number of dead colonies increased during the winter, especially in the Rivers Dun and Itchen. Collecting material generally became more difficult with the onset of winter when many bryozoan colonies degenerated. Live colonies had disappeared completely in some cases by late winter/early spring (on roots in the River Avon and from some roots in the River Itchen) (Table 2). However new growth enabled collection of live colonies in large numbers in spring and summer from all roots in each river (Fig. 1a, Table 2). Accordingly, the mean sizes of live colonies significantly varied over time in all three rivers and were generally smallest in winter (Fig. 1b, Table 3: Test A1). The proportion of colonies with statoblasts was significantly different amongst rivers (Table 3: Test A2). Statoblasts were present in a minority of colonies in the Rivers Avon and Dun and were most commonly observed in late summer and autumn. Statoblasts were almost entirely absent from bryozoans collected from the River Itchen (Table 2).

**Table 2:** The total number of bryozoan colonies collected per river in each sampling trip according to different categories (and the numbers collected from trees 1, 2, and 3). Colony categories are: alive but not producing statoblasts (- stats); alive and producing statoblasts (+ stats); dead with no statoblasts (- stats); dead with mature statoblasts (+ stats). The last four columns provide data for the number (and percentage) of uninfected, covertly and overtly-infected colonies that were identified by dissection and the overtly-infected colonies’ respective sac number. See Methods for further details on sampling to determine infection status

River	Sampling trip	Sampling date	Alive (- stats)	Alive (+ stats)	Dead (- stats)	Dead (+ stats)	Covertly-infected	Uninfected	Overtly-infected	Sac number
Avon	1	20/10/2011	143 (48, 35, 60)	147 (50, 60, 37)	1 (0, 1, 0)	0	6 (19%), 8 (25%), 8 (26%)	25 (81%), 24 (75%), 22 (71%)	0	0
	2	05/12/2011	101 (34, 21, 46)	53 (29, 17, 7)	6 (3, 2, 1)	3 (1, 1, 1)	25 (53%), 25 (68%), 29 (81%)	22 (47%), 12 (32%), 7 (19%)	0	0
	3	19/01/2012	14 (3, 0, 11)	8 (0, 2, 6)	4 (1, 0, 3)	3 (2, 0, 1)	3 (60%), 2 (100%), 14 (78%)	2 (40%), 0, 4 (22%)	0	0
	4	05/03/2012	0	0	0	0	0	0	0	0
	5	18/04/2012	242 (94, 77, 71)	1 (1, 0, 0)	2 (0, 0, 2)	0	13 (41%), 8 (25%), 10 (31%)	19 (59%), 24 (75%), 22 (69%)	0	0
	6	11/06/2012	214 (72, 74, 68)	75 (23, 24, 28)	1 (0, 0, 1)	3 (3, 0, 0)	11 (34%), 20 (59%), 16 (50%)	21 (66%), 14 (41%), 16 (50%)	0, 0, 1 (1%)	0, 0, 2
	7	18/07/2012	227 (81, 81, 65)	51 (11, 11, 29)	4 (0, 1, 3)	1 (0, 0, 1)	14 (44%), 14 (44%), 12 (39%)	18 (56%), 18 (56%), 19 (61%)	0, 1 (1%), 1 (1%)	0, 1, 2
	8	29/08/2012	221 (80, 72, 69)	54 (17, 9, 28)	7 (3, 3, 1)	2 (0, 2, 0)	20 (49%), 11 (31%), 42 (76%)	21 (51%), 24 (69%), 13 (24%)	0, 1 (1%), 2 (2%)	0, 5, 6, 11
Dun	1	25/10/2011	166 (73, 35, 58)	57 (22, 8, 27)	15 (3, 4, 8)	3 (0, 1, 2)	22 (59%), 14 (40%), 15 (52%)	15 (41%), 21 (60%), 14 (48%)	0, 0, 1 (1%)	0, 0, 14
	2	08/12/2011	129 (56, 24, 49)	19 (8, 9, 2)	51 (30, 10, 11)	9 (3, 4, 2)	39 (89%), 32 (91%), 35 (85%)	5 (11%), 3 (9%), 6 (15%)	1 (1%), 2 (5%), 0	2, 2, 1, 0
	3	23/01/2012	96 (37, 26, 33)	9 (2, 3, 4)	48 (32, 7, 9)	10 (5, 3, 2)	39 (89%), 32 (100%), 35 (90%)	5 (11%), 0, 4 (10%)	0	0
	4	08/03/2012	120 (50, 35, 35)	6 (1, 4, 1)	37 (13, 10, 14)	0	23 (72%), 32 (100%), 24 (75%)	9 (28%), 0, 8 (25%)	0	0
	5	23/04/2012	236 (92, 48, 96)	3 (3, 0, 0)	8 (5, 0, 3)	0	6 (19%), 20 (65%), 9 (28%)	26 (81%), 11 (35%), 23 (72%)	1 (1%), 2 (4%), 0	5, 3, 6, 0
	6	06/06/2012	243 (99, 44, 100)	0	5 (0, 5, 0)	0	22 (69%), 28 (88%), 25 (78%)	10 (31%), 4 (13%), 7 (22%)	1 (1%), 0, 0	1, 0, 0
	7	23/07/12	215 (88, 45, 82)	16 (5, 1, 10)	11 (6, 2, 3)	0	9 (28%), 15 (48%), 10 (32%)	23 (72%), 16 (52%), 21 (68%)	3 (3%), 1 (2%), 0	26, 4, 1, 25, 0
	8	05/09/2012	189 (77, 42, 70)	33 (7, 7, 19)	16 (8, 1, 7)	3 (2, 0, 1)	23 (64%), 27 (68%), 33 (75%)	13 (36%), 13 (33%), 11 (25%)	3 (3%), 1 (2%), 1 (1%)	1, 4, 5, 11, 9
Itchen	1	15/10/2012	239 (83, 83, 73)	1 (1, 0, 0)	20 (8, 10, 2)	0	23 (72%), 26 (81%), 21 (66%)	9 (28%), 6 (19%), 11 (34%)	0, 0, 1 (1%)	0, 0, 2
	2	03/12/2012	258 (79, 88, 91)	1 (0, 1, 0)	32 (19, 5, 8)	0	19 (59%), 15 (47%), 16 (50%)	13 (41%), 17 (53%), 16 (50%)	0, 1 (1%), 1 (1%)	0, 2, 1
	3	14/01/2013	87 (84, 1, 2)	0	13 (13, 0, 0)	0	28 (33%), 0, 1 (50%)	56 (67%), 1 (100%), 1 (50%)	0	0
	4	25/02/2013	26 (26, 0, 0)	0	20 (19, 1, 0)	1 (1, 0, 0)	12 (44%), 0, 0	15 (56%), 0, 0	0	0
	5	03/04/2013	11 (0, 0, 11)	0	3 (0, 0, 3)	0	0, 0, 3 (27%)	0, 0, 8 (73%)	0	0
	6	20/05/2013	191 (47, 97, 47)	0	6 (1, 2, 3)	0	1 (3%), 8 (25%), 25 (78%)	31 (97%), 24 (75%), 7 (22%)	0	0
	7	01/07/2013	222 (97, 97, 28)	0	9 (2, 3, 4)	0	11 (34%), 14 (44%), 25 (89%)	21 (66%), 18 (56%), 3 (11%)	0	0
	8	12/08/2013	296 (97, 100, 99)	4 (3, 0, 1)	0	0	14 (44%), 18 (56%), 13 (41%)	18 (56%), 14 (44%), 19	1 (1%), 0, 1 (1%)	1, 0, 12

**Table 3:** Summary of statistical results (and associated Test numbers as identified in the Results section) for analyses of bryozoan hosts and effects of infections on hosts and patterns of covert infections. Statistical results presented for generalised linear modelling (GLM) and generalised linear mixed modelling (GLMM) are presented as Likelihood Ratio Test (LRT) results with the test statistic as *D*

Focus	Test	Statistical test (error distribution)	Test statistic^a^	*df*	*P*	Statistical analysis
Bryozoan hosts	A1	GLM (Quasi-Poisson)	80.683	1	< 0.001	Live colony size varies over time in all three rivers
	A2	GLM (Binomial)	498.68	2	< 0.001	Proportion of statoblast-producing colonies varies amongst rivers
Covert infections	B1	GLM (Binomial)	82.826	2	< 0.001	Proportions vary between rivers
	B2	GLM (Binomial)	22.486	3	< 0.001	Proportions vary over time within rivers
	B3	GLM (Binomial)	40.248	6	< 0.001	Proportions vary amongst roots within rivers
	B4	GLMM (Binomial) Random structure: River/Root/Trip	9.777	1	0.002	Less likely in larger, statoblast-producing colonies
	B5	GLMM (Binomial) Random structure: River/Root/Trip	4.740	1	0.029	More likely in conditions of low productivity, low temperature and high dissolved oxygen in small colonies not producing statoblasts
	B6	GLMM (Gaussian) Random structure: River/Root/Trip	8.194	1	0.004	High infection severity more likely in conditions of low productivity, low temperature and high dissolved oxygen
	B7	GLM (Gaussian)	5.444	1	0.007	Infection severity likely to be reduced in statoblast-producing colonies
	B8	GLM (Gaussian)	2.539	1	0.042	Infection intensity likely to be reduced in statoblast-producing colonies
	B9	GLM (Gaussian)	0.434	1	0.401	Infection intensity not influenced by colony size

^a^The test statistics for Chi-square tests and GLMMs with a binomial error distribution are *χ* ^2^ values

### *Tetracapsuloides bryosalmonae* infections over time and space

**Fig. 2:**
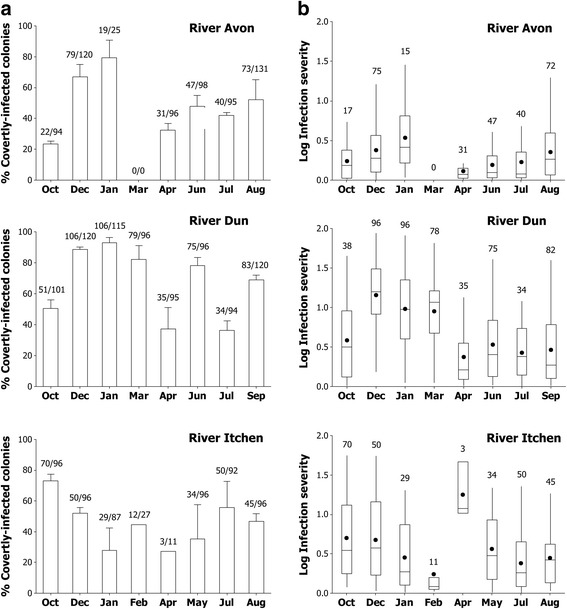
Covert infection prevalence and severity over time. **a** Mean prevalence of covertly-infected colonies sampled from three tree roots every 45 days over 12 months in the Rivers Avon, Dun and Itchen. Bars are one standard error from the mean and numbers are the number of covertly-infected colonies/total number of colonies. **b** Boxplot showing mean (black circle) log infection severity (ng/μl) in the Rivers Avon, Dun and Itchen over time (sampling every 45 days over 12 months). Numbers are *n*-values (excluding dead colonies with statoblasts and null values)

Covert infections were present throughout the year in all three rivers (Fig. 2a). GLM indicated that the proportion of covert infections varied significantly amongst rivers (Table 3: Test B1) and for sampling trips within each river (Table 3: Test B2). In the River Avon the mean prevalence of covert infections was lowest in October (23%) and highest in January (76%). In the River Dun, mean covert infection prevalences were lowest in April (36%) and, as in the Avon, highest in January (92%). In contrast, in the River Itchen mean covert infection prevalences were lowest in January (33%) and highest in October (73%). Covert infection prevalences were also variable amongst roots within rivers (ranging between 3 and 100% at any given time (Table 2, Table 3: Test B3).

Overt infections were rare (detected in 0–5% of colonies per root at a given time) (Table 2) but were present in at least one river in most sampling periods, apart from during January-March, when they were not encountered. The very low prevalences of overt infection precluded any analyses of infection patterns. There is a suggestion that the numbers of sacs increased in late summer in all rivers (Table 2).

### Covert infections in relation to host and environmental conditions

Rivers, roots nested within rivers and sampling trips nested within roots explained a large amount of variation in covert infection status. These variables were therefore included as random effects within subsequent mixed models to assess the significance of host characters on covert infection status (Table 1). The two-way interaction between colony size and statoblast presence had a significant effect on infection status (Table 3: Test B4), with a unit increase in size of colonies containing statoblasts decreasing the likelihood of colonies being covertly-infected by 0.920 times (odds ratio). This result suggests that the utilisation of resources by concomitant covert infections reduces host growth if bryozoans are also investing in statoblast production. Notably there were no other discernible relationships between covert infection and colony size in general nor in the propensity to produce statoblasts.

Rivers, roots nested within rivers and sampling trips nested within roots were also included as random effects in mixed models to assess the significance of environmental variables (summarised by PC1 and PC2 values) on covert infection status in the Rivers Avon and Dun. The three-way interaction between colony size, statoblast presence and PC1 had a significant effect on covert infection status (Table 3: Test B5). An increase in PC1’s positive loadings (mainly productivity and temperature) and a decrease in PC1’s negative loadings (e.g. DO), were associated with an increase in the size of statoblast-producing colonies - in turn this was associated with statoblast-bearing colonies being 0.953 times (odds ratio) less likely to be covertly infected. This pattern mirrors the above results, suggesting an energetic drain caused by covert infections that reduces host growth when bryozoans are also investing in statoblast production.

Covert infection intensity values are provided in Additional file 8: Table S5. The associated infection severities varied over time both within and amongst rivers (Fig. 2b). Mean covert infection severities were enhanced in overwintering colonies in the Rivers Avon and Dun but were highest in April in the River Itchen. Mean infection severity was generally lowest in the River Avon. None of the explanatory variables (Table 1) significantly affected infection severity in mixed models with rivers, roots nested within rivers and sampling trips nested within roots as random effects. However, PC1 had a significant effect on infection severity (Table 3: Test B6), with a unit increase in PC1 causing a decrease in infection severity of -0.237 (± 0.130 SE). In other words, an increase in PC1’s positive loadings (mainly productivity and temperature) and a decrease in PC1’s negative loadings (e.g. DO) decreased covert infection severity. Covert infection severity (analysing data pooled across space and time) was negatively influenced by whether colonies were producing statoblasts, with statoblast presence associated with a -0.206 (± 0.076 SE) decrease in infection severity (Table 3: Test B7). Covert infection intensity was similarly likely to be lower in colonies that were producing statoblasts (Table 3: Test B8) and was not affected by colony size (Table 3: Test B9).

## Discussion

### Patterns of infection in space and time

Relatively few studies have assessed covert infection dynamics in invertebrate populations [2] and most have examined prevalences of viruses in insect, mollusc and crustacean populations over short time periods or at single times in different years (e.g. [8, 9, 39–41]). Our programme of systematic sampling provides evidence that bryozoan colonies are variously present throughout the year and that covert infections of myxozoans are continuously harboured in these bryozoan populations. Sampling populations every 45 days revealed that covert infection prevalences varied between the three rivers, over time within the rivers, and amongst the three populations sampled within each river. Bryozoan colonies became notably harder to collect over the winter period as they shrank in size, but collecting material every 45 days from the same population is likely also to have diminished populations on tree root systems sufficiently to preclude collection in some cases. Thus, we failed to collect bryozoans on one date in late winter from the River Avon and colonies were absent from two tree root systems in the River Itchen in late February and early April. This effect of course would not pertain to populations on other tree roots. As spring commenced colonies became more plentiful and populations collected from all tree roots once again harboured covert infections. It should be noted that adherent statoblasts were likely to be present locally even when colonies were unavailable for collection. Our previous research has demonstrated that substantial proportions (between 14 and 100% in the River Avon and between 33 and 100% in the River Dun; calculated from data in Abd-Elfattah et al. [22]) of statoblasts carry covert infections and that moderate levels of infections can promote hatching [29]. The recruitment of a considerable number of infected progeny should therefore ensue when seasonal conditions improve. Indeed, such recruitment is likely to have contributed to the flush of growth that enabled the collection of colonies from all roots in spring.

Evidence for temporal variation in *T. bryosalmonae* infections in *F. sultana* colonies has also been obtained in the River Cerne (Dorset) by sampling every 3 weeks from April to September [18]. There infection prevalences detected by PCR ranged between 0 and 53% in bryozoan populations on three tree root systems, with highest values occurring from April to early June followed by declines in late summer. The PCR study did not distinguish covert from overt infections, however the proportions of bryozoans harbouring overt infections (presence of sacs) was also determined independently. Overt infection prevalences followed a similar temporal trend (but were delayed by one sampling period) and ranged between 0 and 41%. It can be assumed that covert infections will have contributed to the general prevalences detected by PCR. The levels of covert infections reported here are generally much higher and consistent with prevalences reported for bryozoan populations in the Rivers Avon and Dun that were characterised at different periods by Abd-Elfattah et al. [22]. The disparity in covert infection prevalences in the River Cerne study likely reflect the use of more sensitive primers here and in the study by Abd-Elfattah et al. [22].

### Influence of environmental conditions

Modelling suggests that covert infection strategies may arise if there is seasonal variation in transmission rate or variation in host density [1]. Our results provide some indirect evidence for both of these scenarios. GLMM analyses revealed that covert infections are more likely to occur and to be more severe when BOD, nitrogen, temperature, chloride, orthophosphate, magnesium and coliforms are lower and DO and alkalinity are higher. Although data for environmental conditions were only analysed for the last four sampling periods (beginning in April) in the Rivers Avon and Dun, these results are consistent with the maintenance of covert infections during periods of low productivity and thus poor conditions for both colony growth and the development of overt infections of *T. bryosalmonae* as a result of low host food availability and low temperatures. Accordingly, covert infections were notably common and widespread in winter in the Rivers Avon and Dun when bryozoan density was low.

Our laboratory studies have demonstrated that the growth of *F. sultana* and the development of overt infections of *T. bryosalmonae* are both stimulated by increasing temperatures [19, 28] and food [31]. It is therefore possible that conditions of higher temperatures and productivity may have promoted overt infection development in late spring/early summer as observed in populations in the River Cerne [18] along with the concomitant clearance of infection in a proportion of colonies (e.g. in some 37% of bryozoans from the River Cerne [28]), resulting in a reduced likelihood of covert infections. Although we did not detect overt infections in any appreciable number of colonies, the period of peak overt infection prevalences is relatively short (weeks) and may have been missed by our 45-day sampling regime. Other explanations for the low detection of overt infections include patchy distributions of infections within tree root systems and bryozoan resistance to overt infection development.

### Effects of covert infection

Our field study revealed that larger colonies that were producing statoblasts were less likely to be covertly infected, an effect that does not change with the inclusion of water chemistry in the statistical models. This may indicate that covert infections compromise growth of colonies when they are also investing in statoblast production. Notably, an earlier laboratory study provides support for this scenario. Tops et al. [28] found that growth rates of uninfected statoblast-producing colonies were higher than those of covertly infected statoblast-producing colonies. Their analysis was performed on data collected from bryozoans deriving from a single site over a limited time period and infection times and doses were not controlled for. Nevertheless, the combined laboratory results of Tops et al. [28] and our current field study provide relatively strong evidence for impacts of covert infections on growth when bryozoans are also producing statoblasts. An alternative explanation for the results from our field study is that large statoblast-producing colonies are particularly healthy and resist infection better than smaller colonies. Separating these alternative hypotheses would require controlled laboratory experiments.

As argued above, in general, covert infections appear to have no impact on colony growth, apart from when colonies are investing in statoblast production. Similarly, the ability to produce statoblasts is apparently not compromised by covert infections. Notably, the vast majority of colonies sampled were lacking statoblasts over the course of our study (a total of 3886 colonies lacked statoblasts compared to a total of 538 colonies with statoblasts; see Alive (-stats) and Alive (+stats) in Table 2). This suggests that the negative effects of covert infections may be relatively rarely experienced by bryozoans at any one time in populations. Furthermore, covert infections have been shown to improve statoblast hatching [29], a finding that may partly result from a trade-off between statoblast hatching and maternal colony growth. Clearly, however, in order to fully understand the overall effects of covert infections and associated host-parasite interactions, detailed and controlled studies across the bryozoan life-cycle are required. These should include exposing bryozoans from a range of sites to variation in environmental conditions and characterising growth, statoblast and larval development, recruitment from these propagules, and the fate and contributions of colony fragments.

### Covert infections, parasite persistence and disease reservoirs

The ability of *T. bryosalmonae* to regress or remain as non-virulent covert infections during sub-optimal conditions for bryozoan hosts, coupled with vertical transmission to asexually produced offspring (statoblasts) [22] and to colony fragments [29, 42], provide a collective means of both ensuring and amplifying persistent infections in clonal bryozoan hosts. We demonstrate here that covert infections are often supported by more than 50% of the bryozoan colonies in local populations. In addition, our results imply that a relatively small proportion (some 14% overall) of the covertly-infected bryozoan population experiences negative effects of covert infection, i.e. reduced growth when simultaneously investing in statoblast production, and we note that producing statoblasts is a transitory state. Furthermore, results from another study [29] suggest that this negative effect may be traded off against enhanced statoblast hatching. Bryozoan populations therefore appear to represent persistent disease-agent reservoirs for salmonid fish. Given the appropriate environmental cues, these covert infections can be expected to convert to overt infections, enabling horizontal transmission to local farmed and wild fish. The presence, persistence and impacts of this disease reservoir are demonstrated by recurrent annual outbreaks of PKD in which up to 100% of farmed fish can be infected [21] and the potential for serious disease problems in wild fish populations [43, 44].

### Comparative biology of covert infections in invertebrate hosts

Collective research suggests that malacosporeans commonly employ covert infection strategies in bryozoan hosts. The repeated appearance and disappearance of sacs of *T. bryosalmonae* in colonies of *F. sultana* [18, 28, 45] and of *Buddenbrockia allmani* in colonies of *Lophopus crystallinus* [20] in laboratory culture provide graphic demonstration of the retention of cryptic stages during covert infection. It is clear that covert infections can be widespread and commonly reach prevalences exceeding 50% in populations of both *F. sultana* and *L. crystallinus* ([20]; data reported here). Covert infection prevalences of malacosporeans forming vermiform stages in bryozoan populations remain unknown. The high infection prevalences of sac-forming malacosporeans are explained by transmission of infection by malacospores released from intermediate fish hosts and by ‘vertical’ transmission of covert infections to colony fragments [20, 29] and statoblasts [20, 22]. We know relatively little about transmission from fish although pilot studies demonstrate that this is achieved over a relatively brief period of time to a minority of colonies, i.e. ~15% (6 of 41 colonies) and ~8% (1 of 12 colonies) for the bryozoans, *F. sultana* and *Plumatella fungosa* over 4 and 2 week periods, respectively (Table 2) [25]. Substantial contributions from vertical transmission are therefore implicated and these should facilitate both the persistence and amplification of infection in local populations. There is a body of evidence for transport of statoblasts by waterfowl (e.g. [46–48]). Because covert infections are present in many statoblasts, can enhance statoblast hatching [29] and result in overt infections that are transmissive to fish [22], such transport is likely to introduce malacosporean infections to new sites. Infection of a larva of *F. sultana* by *T. bryosalmonae* has been demonstrated by PCR (Fontes unpub. data). However, the rarity of larval production in some *F. sultana* populations suggests this form of transmission makes a minor contribution to malacosporean infection persistence.

There is little evidence that covert infections of *T. bryosalmonae* are the result of host suppression of overt infection development [29]. Hosts experiencing poor conditions (such as low food levels) are often immunocompromised [49], yet covert infections are particularly linked with such unproductive conditions while overt infections develop during favourable conditions when bryozoan hosts are vigorously growing [28, 31]. In addition, we have experimentally shown that overt infections develop when very low food levels promote terminal investment [29], thus providing further evidence that bryozoans are unable to suppress infection during adverse conditions.

The adoption of covert infection strategies by malacosporeans begs the question of whether periods of covert infection are also employed by other parasites of invertebrate hosts. Here we discuss this question specifically regarding their myxozoan relatives - the myxosporeans, whose life-cycles involve annelids as primary hosts [50]. This focus enables us to pursue comparative insights and thus to highlight key questions about covert infection strategies, impacts and effects. Because covert infections have been characterised in a range of invertebrate hosts [2], many of these questions are likely to be equally relevant to covert infections in other parasite-invertebrate host systems.

We are aware of one study that provides evidence for a myxosporean undergoing periods of covert infection. Gilbert & Granath [15] found that actinospores of *Myxobolus cerebralis* were released in faeces of laboratory-infected *Tubifex tubifex* in a cyclical manner, approximately 12 times over a 59-day period. This translated to individual worms releasing actinospores over several consecutive days punctuated by periods of no release for a week or more. They also found that infections could persist for up to 10 months after actinospore shedding had ceased, that actinospore release could be resumed some 20 months after worms were first infected and that individual worms can remain persistently infected throughout their lifespan. Similar results for periodic actinospore shedding were obtained for worms naturally infected in the field. These findings provide evidence that persistent covert infections of *M. cerebralis* may optimise transmission to fish by periodically converting to overt infections over the multiyear year life span of worm hosts (e.g. up to 3 years [51]). The potential for persistent covert infections of *M. cerebralis*, however, is apparently not linked with substantial infection prevalences in the field. PCR-based assays demonstrate that *M. cerebralis* prevalences in *T. tubifex* populations are usually < 10% and typically < 1% (e.g. [50, 52, 53]). Whether such covert infections are generally avirulent like those characterised for malacosporeans in bryozoan hosts remains unknown. During periods of overt infection both malacosporeans and myxosporeans are virulent, variously causing reduction in host growth, reproduction (statoblasts in bryozoan hosts) and mortality [28, 31, 54–57].

Morris & Adams [42] showed that infections of an unidentified myxosporean can be transmitted when the oligochaete host, *Lumbriculus variegatus*, undergoes fission by architomy (the fragmentation of the body at a particular point into individual or groups of segments that then regenerate the missing tissues of the new individuals; [58]). Similarly, Atkinson & Bartholomew [59] report that the myxosporean *Myxobilatus gasterostei* transmits during paratomy (the fragmentation of the body perpendicularly to the antero-posterior axis following internal tissue reorganisation) of its oligochaete host, *Nais communis*. Whether myxosporean infections in other annelids are transmitted during fission, which has evolved multiple times in annelids [60], remains unknown. Covert infection strategies evidently do not depend on the potential for vertical transmission to products of fission since *M. cerebralis* undergoes bouts of covert infection in *T. tubifex*, a species that does not undergo fission. Various oligochaetes in freshwater habitats are known to form cysts that survive adverse conditions [61], including *T. tubifex* [62], but whether encysted worms harbour covert infections has not been investigated. It is also unknown if covert infections of myxosporeans are vertically transmitted to eggs. Infection of dormant cysts and eggs might enable large-scale dispersal of myxosporeans, contributing to the dispersal that is likely to be achieved by ingestion of infected fish by piscivorous birds [63].

Baxa et al. [64] provide evidence that some myxosporean covert infections may result from suppression by annelid hosts. In their infection trial, clonal lines deriving from different genetic lineages of *T. tubifex* were established in the laboratory and exposed to infection of *M. cerebralis* myxospores. Actinospores were released in varying numbers by clonal lines from different genetic lineages, but arrested development was revealed by histological examination and in situ hybridisation in clonal lines deriving from a particular genetic lineage characterised by random amplification of polymorphic DNA (RAPD) PCR analysis. Early stages of development were present but actinospores were never shed by worms of this lineage. As far as we know, the extent that such arrested development may explain differing susceptibilities to infection associated with genetically distinct lineages of annelid hosts has not been systematically addressed. Nor, to our knowledge, is it known whether infection can be transferred to fish hosts via ingestion of ‘resistant’ worms that harbour covert infections but suppress actinospore development. Another explanation is that hyperparasitism may preclude myxosporean development. Morris & Freeman [65] found that co-infection by the microsporidian *Neoflabelliforma aurantiae* results in hyperparasitism of concurrent myxosporean infections in oligochaete worms and provide evidence for the cessation of development and release of actinospores caused by hyperparasitism. Whether such suppressed myxosporean infections are capable of subsequent development and future transmission if microsporidian infections are lost remains unknown.

The following list of outstanding questions about the biology of covert infections is inspired by our specific contrasts between malacosporeans and myxosporeans. Studies addressing these questions will increase our understanding of the evolutionary ecology of parasite-invertebrate host interactions and the contexts within which covert infection strategies may evolve and are maintained in hosts with varying life histories. They are also relevant to the general issue of how covert infection dynamics may result in disease reservoirs.

- How common are covert infections in invertebrates?
- What are the general impacts of covert infections on host fitness?
- Are fission and fragmentation by invertebrate hosts common routes for covert infection transmission?
- Are fission and fragmentation linked with the evolution of covert infection strategies?
- What environmental cues provoke covert infections to develop into overt infections?
- Does cycling between covert and overt infections characterise covert infection strategies?
- Does host capacity to undergo encystment and to produce resting stages select for covert infection strategies?
- How common is vertical transmission of covert infections to sexual offspring?
- Do covert infections contribute to parasite dispersal?
- How generally do covert infections contribute to the maintenance and spread of disease reservoirs?
- Can covert infections commonly result from immunosuppression?
- Do covert infections increase the probability of outcrossing for parasites?
- Are covert infections protective to invertebrate hosts, conferring immunity to subsequent infection (‘superinfection’)?
- How do covert infection strategies vary in parasites with simple (direct) and complex (indirect) life-cycles?
- How commonly do co-infections suppress development causing apparent covert infection and is such developmental suppression permanent?

## Conclusions

To our knowledge, this is the first long-term study of covert infections in a field setting and therefore provides unique insights into what is increasingly being recognised as a common infection strategy [1]. By addressing a series of hypotheses, we demonstrate that covert infections are widespread and persist over space and time in bryozoan populations. We found variation in prevalences within and between rivers, at least some of which may be attributed to variation in environmental conditions (e.g. productivity), host density and size. The persistence of infected adult colonies and parasite transmission via statoblasts may maximise horizontal transmission to fish hosts over time by establishing a reservoir that poses a long-term risk of disease outbreaks for farmed fish and wild salmonids. This risk may be particularly exacerbated by environmental change [2, 19]. A review of covert infections caused by myxozoans in annelid and bryozoan hosts suggests that covert infection strategies in different systems may share common features. The implications of covert infections are substantial in terms of understanding host-parasite interactions and the impacts of environmental change, and prompt us to identify several outstanding questions regarding the biology of covert infections.

## Additional files

Additional file 1: Table S1. Site locations. (DOCX 12 kb)

Additional file 2: Table S2. Environmental variables data. (DOCX 14 kb)

Additional file 3: Detailed CTAB protocol including modifications to the original protocol. (DOCX 18 kb)

Additional file 4: Figure S1. Water temperature. Temperature measurements over 12 months according to the 8 sampling trips every 45 days for each river. (TIFF 235 kb)

Additional file 5: Table S3. Water flow data. (DOCX 13 kb)

Additional file 6: Figure S2. PCA plot. Principal components analysis (PCA) scores for environmental variables. Ellipses are normal contour lines with probability of 68% done by cluster analysis of rivers. Data points for each river are coloured by sampling trip (River Avon: 5th trip - 18/04/12; 6th trip - 11/06/12; 7th trip - 18/07/12; 8th trip - 29/08/12; River Dun: 5th trip - 23/04/12; 6th trip - 06/06/12; 7th trip - 23/07/12; 8th trip - 05/09/12). Variables with vectors pointing in the same direction have similar responses. Points that are close together correspond to observations that have similar scores on the components. PC1 explains 56.0% of the variation. PC2 explains 25.4% of the variance. (TIFF 1262 kb)

Additional file 7: Table S4. Summary of PCA analysis on environmental variables. (DOCX 13 kb)

Additional file 8: Table S5. Colony infection intensity. (DOCX 13 kb)

## Abbreviations

AIC: Akaike information criterion
BOD: Biochemical oxygen demand
CFU: Colony forming units
COD: Chemical oxygen demand
DO: Dissolved oxygen
EA: Environment agency
FTU: Formazin turbidity unit
GLM: Generalised linear model
GLMM: Generalised linear mixed models
GV: Granulovirus
HIV: Human immunodeficiency virus
LRT: Likelihood ratio test
ML: Maximum likelihood estimation
NLS: National laboratory service
PC: Principal component
PCA: Principal components analysis
PKD: Proliferative kidney disease
RAPD: Random amplification of polymorphic DNA
REML: Restricted maximum likelihood estimation
SE: Standard error
SVD: Singular value decomposition
