# Supplementary material for: Persistence, impacts and environmental drivers of covert infections in invertebrate hosts
Source: Parasit Vectors. 2017 Nov 2;10:542. doi: 10.1186/s13071-017-2495-8 (PMC5668978; doi:10.1186/s13071-017-2495-8)
Supplement: Supplementary file 1 — Site locations. (DOCX 12 kb) [file 13071_2017_2495_MOESM1_ESM.docx]

**Additional file 1: Table S1.** Geographical locations of the roots sampled in each river system according to latitude and longitude (in WGS84) and ordnance survey (OS) grid references.

| **River name** | **County** | **Tree root**  **system** | **Latitude** | **Longitude** | **OS Grid Ref** |
| --- | --- | --- | --- | --- | --- |
| Avon | Hampshire | 1 | 50°54′50.03″N | 001°47′36.02″W | SU 1462512735 |
|  |  | 2 | 50°54′50.23″N | 001°47′36.63″W | SU 1461312741 |
|  |  | 3 | 50°54′48.15″N | 001°47′33.26″W | SU 1467912677 |
| Dun | Berkshire | 1 | 51°24′56.83″N | 001°30′32.97″W | SU 3423168644 |
|  |  | 2 | 51°24′54.61″N | 001°30′29.83″W | SU 3429268576 |
|  |  | 3 | 51°24′49.72″N | 001°30′20.31″W | SU 3447768426 |
| Itchen | Hampshire | 1 | 51°05′19.78″N | 001°17′12.33″W | SU 50053241 |
|  |  | 2 | 51°05′12.07″N | 001°17′22.21″W | SU 49863217 |
|  |  | 3 | 51°03′20.90″N | 001°18′48.66″W | SU 48212872 |
