# Supplementary material for: Persistence, impacts and environmental drivers of covert infections in invertebrate hosts
Source: Parasit Vectors. 2017 Nov 2;10:542. doi: 10.1186/s13071-017-2495-8 (PMC5668978; doi:10.1186/s13071-017-2495-8)
Supplement: Supplementary file 2 — Environmental variables data. (DOCX 14 kb) [file 13071_2017_2495_MOESM2_ESM.docx]

**Additional file 2: Table S2.** Results for the environmental variables collected per river and respective sampling date. Parameter values presented as “<n” were converted to zeros for statistical analyses (ammonia, ammoniacal nitrogen, orthophosphate, biological oxygen demand and chlorophyll-a) and parameters with very low variation or a lot of missing data were excluded (chemical oxygen demand and cadmium).

| **River** | **Sampling**  **trip** | **Dissolved oxygen (mg/l)** | **Chemical Oxygen Demand (mg/l)** | **pH** | **Alkalinity (mg/l)** | **Hardness (mg/l)** | **Ammonia (mg/l)** | **Ammoniacal Nitrogen (mg/l)** | **Nitrate (mg/l)** | **Nitrite (mg/l)** | **Nitrogen (mg/l)** | **Orthophosphate (mg/l)** | **Conductivity (µS/cm)** | **Turbidity (FTU)** | **Magnesium (mg/l)** | **Calcium (mg/l)** | **Chloride (mg/l)** | **Bacterial Oxygen Demand (mg/l)** | **Chlorophyll a (µg/l)** | **Confirmed coliforms (CFU/0.1l)** | **Presumptive coliforms (CFU/0.1l)** | **Cadmium (mg/l)** |
| --- | --- | --- | --- | --- | --- | --- | --- | --- | --- | --- | --- | --- | --- | --- | --- | --- | --- | --- | --- | --- | --- | --- |
| Avon | 18/04/12 | 9.05 | <10 | 8.02 | 202 | 244.07 | 0.0036 | 0.186 | 5.351 | 0.059 | 5.41 | 0.028 | 535 | 9.1 | 2.09 | 94.2 | 21.3 | 2.70 | 17.9 | 436 | 1091 | <0.1 |
|  | 11/06/12 | 7.70 | 12 | 7.89 | 221 | 268.90 | 0.0071 | 0.427 | 5.350 | 0.081 | 5.43 | 0.094 | 568 | 2.9 | 2.17 | 104.0 | 19.6 | 1.40 | 3.7 | 3700 | 3700 | <0.1 |
|  | 18/07/12 | 6.77 | 10 | 7.87 | 231 | 284.39 | 0.0052 | 0.262 | 5.142 | 0.089 | 5.23 | 0.109 | 586 | 2.9 | 2.29 | 110.0 | 19.9 | 1.40 | 2.0 | 21600 | 24000 | <0.1 |
|  | 29/08/12 | 7.24 | <10 | 7.95 | 226 | 263.41 | 0.0044 | 0.187 | 5.247 | 0.053 | 5.30 | 0.079 | 564 | 2.5 | 2.05 | 102.0 | 19.6 | 1.30 | 2.3 | 18000 | 20000 | <0.1 |
| Dun | 23/04/12 | 12.54 | 11 | 7.93 | 229 | 272.75 | <0.0005 | <0.03 | 4.735 | 0.005 | 4.74 | <0.02 | 562 | 3.1 | 1.89 | 106.0 | 16.9 | 1.30 | 23.7 | 280 | 280 | <0.1 |
|  | 06/06/12 | 11.30 | 10 | 8.05 | 244 | 285.33 | <0.0007 | <0.03 | 5.041 | 0.009 | 5.05 | <0.02 | 578 | 3.4 | 1.91 | 111.0 | 16.2 | <3.00 | 27.7 | 170 | 189 | <0.1 |
|  | 23/07/12 | 11.15 | <10 | 8.06 | 251 | 293.12 | <0.0007 | <0.03 | 4.947 | 0.013 | 4.96 | 0.040 | 595 | 3.0 | 1.98 | 114.0 | 17.6 | <1.00 | <0.5 | 243 | 270 | <0.1 |
|  | 05/09/12 | 10.19 | <10 | 8.00 | 246 | 287.59 | <0.0007 | <0.03 | 5.109 | 0.011 | 5.12 | 0.041 | 593 | 1.9 | 1.85 | 112.0 | 16.9 | <1.00 | 1.3 | 360 | 360 | <0.1 |
| Itchen | 03/04/13 | 6.00 | - | - | - | 297.87 | - | <0.03 | - | - | 7.56 | 0.024 | - | 2.3 | 1.92 | 116.0 | 18.7 | 1.38 | 3.5 | 54 | 54 | <0.1 |
|  | 20/05/13 | 4.90 | - | - | - | - | - | <0.03 | - | - | 6.75 | 0.028 | - | 2.5 | - | - | 17.9 | 1.10 | 10 | 220 | 220 | <0.1 |
|  | 01/07/13 | 4.80 | - | - | - | 282.95 | - | <0.03 | - | - | 6.22 | 0.049 | - | 3.3 | 1.94 | 110.0 | 18.0 | 1.40 | 3.8 | 522 | 580 | <0.1 |
|  | 12/08/13 | 6.83 | - | - | - | - | - | <0.03 | - | - | 6.36 | 0.039 | - | 1.6 | - | - | 17.5 | <1.00 | 2.7 | 660 | 660 | <0.1 |
