# Supplementary material for: Persistence, impacts and environmental drivers of covert infections in invertebrate hosts
Source: Parasit Vectors. 2017 Nov 2;10:542. doi: 10.1186/s13071-017-2495-8 (PMC5668978; doi:10.1186/s13071-017-2495-8)
Supplement: Supplementary file 3 — Detailed CTAB protocol including modifications to the original protocol. (DOCX 18 kb) [file 13071_2017_2495_MOESM3_ESM.docx]

**Additional file 3**

**CTAB protocol**

DNA was extracted from bryozoan colonies using a modified hexadecyltrimethylammonium bromide (CTAB) protocol based on Winnepenninckx et al. [1] which is described below (for the extraction of two 96 deep well plates, max 192 samples):

1. Add three 3 mm borosilicate glass beads into each well of two 96 deep well plates (Axygen^®^ round bottom 2 ml polypropylene sterile plate #P-DW-20-C)
2. Put bryozoan colonies into each well
3. Add 50 μl of CTAB extraction buffer (2% CTAB, 1.4 M NaCl, 20 mM EDTA, 100 mM Tris-HCl) (**MC**)
4. Grind samples using a TissueLyser II (QIAGEN) four times for 1 min at 30 Hz in the four possible plate positions
5. Spin down at 4000 rpm
6. Add 250 µl of CTAB:β-mercaptoethanol (2% CTAB, 1.4 M NaCl, 20 mM EDTA, 100 mM Tris-HCl; 0.2% β-mercaptoethanol) (**MC**)
7. Incubate at 60°C, 900 rpm for 2 h or overnight (**TS**)
8. Spin down at 4000 rpm
9. Add 10 µl of Proteinase K (2 mg/ml)
10. Spin down at 4000 rpm
11. Incubate at 37°C, 900 rpm for 1 h or overnight (**TS**)
12. Spin down at 4000 rpm
13. Add 100 µl of TE buffer (10 mM Tris-HCl; 1 mM EDTA; pH 8.0) (**MC**)
14. Add 400 µl of Chloroform:Isoamyl alcohol (24:1) (**DT**)
15. Close plates well and mix by inverting several times
16. Spin for 10 min at 4000 rpm
17. Take supernatant (~ 100 µl) to new plate
18. Add 600 µl ice-cold 100% Ethanol (**DT**)
19. Add 30 µl Sodium Acetate (3 M, pH 5.2) (**DT**)
20. Close plates well and mix by inverting several times
21. Precipitate in a -20°C freezer for 1 h or overnight
22. Spin for 25 min at 4000 rpm, 4°C
23. Wash pellets twice:
    1. Remove supernatant by inverting the plate onto sink and dabbing on clean tissue paper
    2. Add 600 µl ice-cold 70% EtOH (**DT**)
    3. Spin for 10 min at 4000 rpm
24. Dry pellets for 30 min at high speed on a Savant^™^ DNA SpeedVac^™^ Concentrator (Thermo Fisher Scientific) with a deep well plate rotor
25. Add 50µl TE (**MC**)
26. Vortex plates well (**TS**)
27. Spin down at 4000 rpm
28. Leave overnight in a fridge for pellets to dissolve
29. Put in a -20°C freezer.

**Notes:**

- **MC** = Use a pipetting reservoir and a 8-channel pipette.
- **DT** = Use DistriTips^®^ syringes on a Distriman^®^ pipette (Gilson^™^).
- **TS** = Use a device such as TS-DW, a Thermo–Shaker for deep well plates (Biosan) that allows simultaneous shaking and incubation.
- During DNA extractions use Impermamat sealing mats throughout (Axygen^®^ chemical resistant silicone 96 round well sealing mat for deep well plates #AM-2ML-RD-IMP), except when using an incubator in which case adhesive PCR plate foils should be used instead.
- For storage of eluted DNA, seal plates using AxyMats^™^ sealing mats (Axygen^®^ 96 round well sealing mat for deep well plate #AM-2ML-RD-S).
- We suggest using yellow tinted pipette tips and a lamp behind plates when transferring supernatant from one deep well plate to another.

**Reference**

1. Winnepenninckx B, Backeljau T, Dewachter R. Extraction of high molecular weight DNA from molluscs. Trends Genet. 1993;9(12):407.
