# Supplementary figures and images for: Persistence, impacts and environmental drivers of covert infections in invertebrate hosts
Source: Parasit Vectors. 2017 Nov 2;10:542. doi: 10.1186/s13071-017-2495-8 (PMC5668978; doi:10.1186/s13071-017-2495-8)

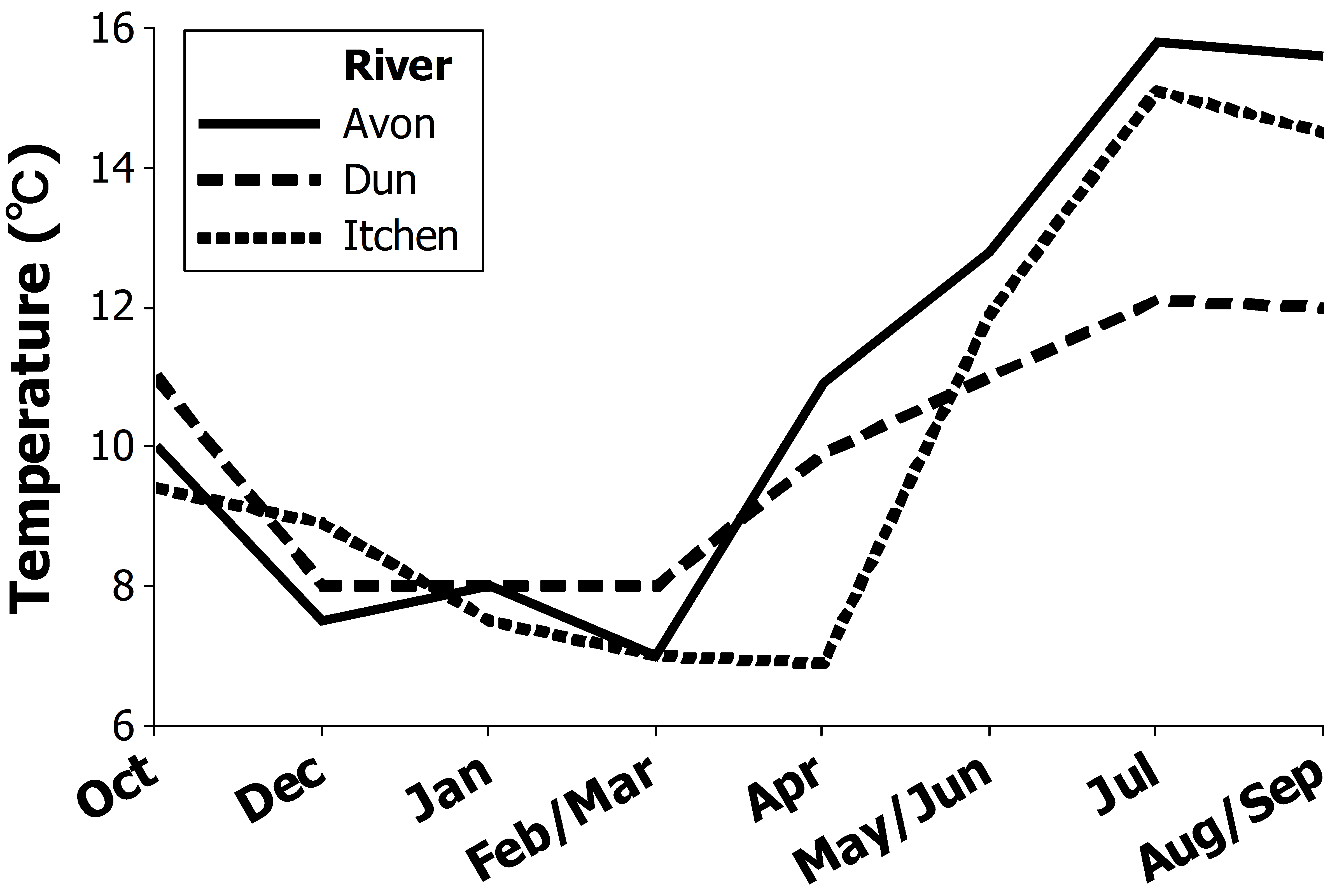

Supplement: Supplementary file 4 — Water temperature. Temperature measurements over 12 months according to the 8 sampling trips every 45 days for each river. (TIFF 235 kb) [file 13071_2017_2495_MOESM4_ESM.tif]

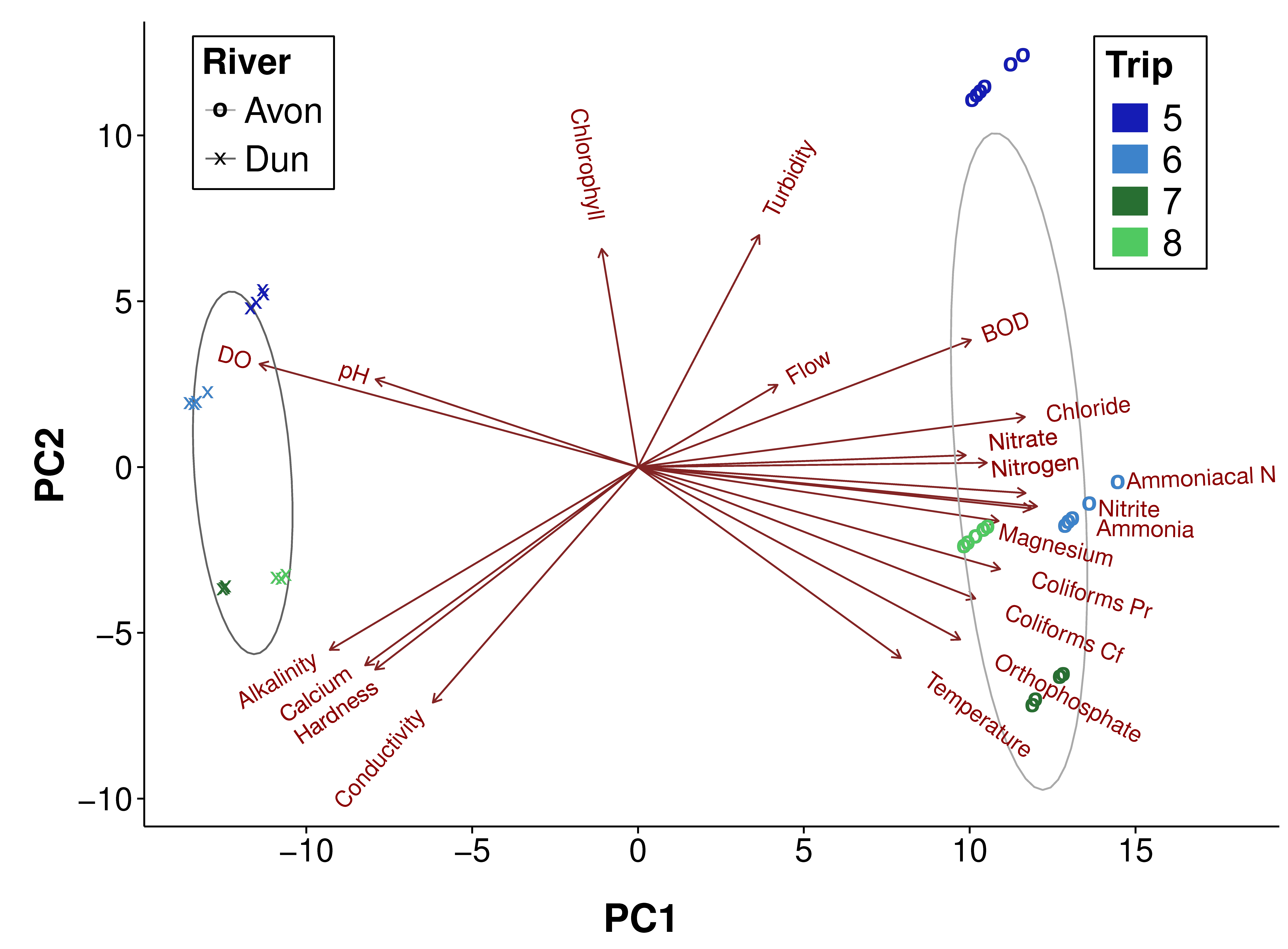

Supplement: Supplementary file 6 — PCA plot. Principal components analysis (PCA) scores for environmental variables. Ellipses are normal contour lines with probability of 68% done by cluster analysis of rivers. Data points for each river are coloured by sampling trip (River Avon: 5th trip - 18/04/12; 6th trip - 11/06/12; 7th trip - 18/07/12; 8th trip - 29/08/12; River Dun: 5th trip - 23/04/12; 6th trip - 06/06/12; 7th trip - 23/07/12; 8th trip - 05/09/12). Variables with vectors pointing in the same direction have similar responses. Points that are close together correspond to observations that have similar scores on the components. PC1 explains 56.0% of the variation. PC2 explains 25.4% of the variance. (TIFF 1262 kb) [file 13071_2017_2495_MOESM6_ESM.tiff]
