# Supplementary material for: Persistence, impacts and environmental drivers of covert infections in invertebrate hosts
Source: Parasit Vectors. 2017 Nov 2;10:542. doi: 10.1186/s13071-017-2495-8 (PMC5668978; doi:10.1186/s13071-017-2495-8)
Supplement: Supplementary file 5 — Water flow data. (DOCX 13 kb) [file 13071_2017_2495_MOESM5_ESM.docx]

**Additional file 5: Table S3.** Flow speed measurements over 12 months according to the 8 sampling trips every 45 days for each river. Flow was not measured in Rivers Avon and Dun on the first sampling trip (indicated by “–”). Flow data are reported as the average speed (n = 3, based on recordings from each root) and associated standard deviation (SD).

| **River** | **Sampling**  **trip** | **Date** | **Mean flow speed**  **(m/s) ± SD** |
| --- | --- | --- | --- |
| Avon | 1 | 20/10/11 | - |
|  | 2 | 05/12/11 | 0.23 ± 0.11 |
|  | 3 | 19/01/12 | 0.23 ± 0.07 |
|  | 4 | 05/03/12 | 0.21 ± 0.15 |
|  | 5 | 18/04/12 | 0.18 ± 0.19 |
|  | 6 | 11/06/12 | 0.15 ± 0.16 |
|  | 7 | 18/07/12 | 0.07 ± 0.11 |
|  | 8 | 29/08/12 | 0.09 ± 0.05 |
| Dun | 1 | 25/10/11 | - |
|  | 2 | 08/12/11 | 0.06 ± 0.03 |
|  | 3 | 23/01/12 | 0.04 ± 0.04 |
|  | 4 | 08/03/12 | 0.04 ± 0.05 |
|  | 5 | 23/04/12 | 0.07 ± 0.06 |
|  | 6 | 06/06/12 | 0.07 ± 0.07 |
|  | 7 | 23/07/12 | 0.01 ± 0.01 |
|  | 8 | 05/09/12 | 0.06 ± 0.03 |
| Itchen | 1 | 15/10/12 | 0.28 ± 0.13 |
|  | 2 | 03/12/12 | 0.44 ± 0.14 |
|  | 3 | 14/01/13 | 0.54 ± 0.27 |
|  | 4 | 25/02/13 | 0.33 ± 0.13 |
|  | 5 | 03/04/13 | 0.39 ± 0.25 |
|  | 6 | 20/05/13 | 0.36 ± 0.12 |
|  | 7 | 01/07/13 | 0.34 ± 0.14 |
|  | 8 | 12/08/13 | 0.32 ± 0.11 |
