# Supplementary material for: Persistence, impacts and environmental drivers of covert infections in invertebrate hosts
Source: Parasit Vectors. 2017 Nov 2;10:542. doi: 10.1186/s13071-017-2495-8 (PMC5668978; doi:10.1186/s13071-017-2495-8)
Supplement: Supplementary file 7 — Summary of PCA analysis on environmental variables. (DOCX 13 kb) [file 13071_2017_2495_MOESM7_ESM.docx]

**Additional file 7: Table S4.** Summary of principal components analysis (PCA) on environmental variables collected from the Rivers Avon and Dun on sampling trips 5 - 8. Results presented include each component’s eigenvalue, proportion of variance, and cumulative variance as well as the loadings for each environmental variable.

|  | | **PC1** | **PC2** |
| --- | --- | --- | --- |
| **Eigenvalue** | | 3.43 | 2.31 |
| **Proportion of variance (%)** | | 56.05 | 25.36 |
| **Cumulative variance (%)** | | 56.05 | 81.41 |
| **Variables** | Flow | 0.10 | 0.13 |
|  | Temperature | 0.18 | -0.30 |
|  | Dissolved Oxygen | -0.27 | 0.16 |
|  | Bacterial Oxygen Demand | 0.23 | 0.20 |
|  | Alkalinity | -0.22 | -0.28 |
|  | Ammonia | 0.28 | -0.06 |
|  | Ammoniacal nitrogen | 0.27 | -0.04 |
|  | Chloride | 0.27 | 0.08 |
|  | Nitrate | 0.23 | 0.02 |
|  | Nitrite | 0.28 | -0.06 |
|  | Total nitrogen | 0.24 | 0.01 |
|  | Orthophosphate | 0.23 | -0.27 |
|  | Chlorophyll a | -0.03 | 0.34 |
|  | Conductivity | -0.14 | -0.37 |
|  | pH | -0.18 | 0.14 |
|  | Turbidity | 0.09 | 0.36 |
|  | Hardness | -0.18 | -0.31 |
|  | Calcium | -0.19 | -0.31 |
|  | Magnesium | 0.25 | -0.08 |
|  | Coliforms (confirmed) | 0.24 | -0.20 |
|  | Coliforms (presumptive) | 0.25 | -0.16 |
