# Supplementary material for: Persistence, impacts and environmental drivers of covert infections in invertebrate hosts
Source: Parasit Vectors. 2017 Nov 2;10:542. doi: 10.1186/s13071-017-2495-8 (PMC5668978; doi:10.1186/s13071-017-2495-8)
Supplement: Supplementary file 8 — Colony infection intensity. (DOCX 13 kb) [file 13071_2017_2495_MOESM8_ESM.docx]

**Additional file 8: Table S5.** Mean colony infection intensity (ng/µl) (± SD and with n values in brackets) of covertly-infected bryozoan colonies over time (sampling every 45 days over 12 months) in the Rivers Avon, Dun and Itchen, excluding dead colonies with statoblasts and null values.

| **River** | **Sampling**  **trip** | **Date** | **Mean infection intensity**  **(ng/µl) ± SD (n)** |
| --- | --- | --- | --- |
| Avon | 1 | 20/10/11 | 8.17 ± 8.97 (17) |
|  | 2 | 05/12/11 | 9.69 ± 15.10 (75) |
|  | 3 | 19/01/12 | 12.00 ± 18.64 (15) |
|  | 4 | 05/03/12 | - |
|  | 5 | 18/04/12 | 3.22 ± 4.07 (31) |
|  | 6 | 11/06/12 | 5.96 ± 6.78 (47) |
|  | 7 | 18/07/12 | 6.84 ± 11.58 (40) |
|  | 8 | 29/08/12 | 14.25 ± 20.67 (72) |
| Dun | 1 | 25/10/11 | 15.43 ± 15.52 (38) |
|  | 2 | 08/12/11 | 41.47 ± 25.61 (96) |
|  | 3 | 23/01/12 | 28.53 ± 23.79 (96) |
|  | 4 | 08/03/12 | 25.73 ± 16.79 (78) |
|  | 5 | 23/04/12 | 7.24 ± 15.50 (35) |
|  | 6 | 06/06/12 | 15.79 ± 19.71 (75) |
|  | 7 | 23/07/12 | 8.70 ± 9.08 (34) |
|  | 8 | 05/09/12 | 12.30 ± 16.62 (82) |
| Itchen | 1 | 15/10/12 | 18.60 ± 19.69 (70) |
|  | 2 | 03/12/12 | 17.46 ± 17.25 (50) |
|  | 3 | 14/01/13 | 6.82 ± 8.61 (29) |
|  | 4 | 25/02/13 | 1.61 ± 3.31 (11) |
|  | 5 | 03/04/13 | 45.29 ± 1.64 (3) |
|  | 6 | 20/05/13 | 12.67 ± 12.95 (34) |
|  | 7 | 01/07/13 | 6.46 ± 8.82 (50) |
|  | 8 | 12/08/13 | 16.13 ± 20.75 (45) |
